# Large Rhinolith Mimicking Atypical Odontogenic Pain: Case Report and Brief Review of the Literature

**DOI:** 10.1155/2021/5550187

**Published:** 2021-04-17

**Authors:** Zahra Vasegh, Mitra Ghazizadeh Ahsaie

**Affiliations:** Department of Oral and Maxillofacial Radiology, School of Dentistry, Shahid Beheshti University of Medical Sciences, Tehran, Iran

## Abstract

Rhinoliths are rare calcified entities in the nasal fossa, frequently originating around a nidus near the midway point in the inferior meatus, where the passage is the narrowest. They can be clinically asymptomatic and undetected for years. In this study, we present a rare case of large rhinolith mimicking atypical odontogenic pain in a 40-year-old Caucasian female in the left nasal cavity. The lesion was detected in the CBCT and removed uneventfully.

## 1. Introduction

Rhinolith is an uncommon calcification in the nasal fossa. Calcareous concretions that occur in the nose arise from the slow accumulation of inflammatory mineral salts such as calcium phosphate, calcium carbonate, and magnesium, around a nidus [1]. The nidus may be endogenous, mostly in adults, or exogenous (e.g., coins, beads, and seeds), mostly in children [2]. The route of entry of the foreign body into the nasal fossa is usually from the anterior; however, in rare cases, the foreign body enters the nose from posterior following sneezing. Rhinoliths are seen in different shapes and sizes, depending on what the nature of the nidus is [3]. The common signs and symptoms following this entity are pain in the maxillofacial region, toothache, headache, epistaxis, fetid odor sensation, nasal discharge, anosmia, and oral halitosis [4]. However, some cases are completely asymptomatic, and the patient is not aware of the entity unless it is find as an incidental finding in radiography. CBCT is helpful for better detecting the location and extent of the rhinolith three dimensionally, especially if surgery is needed. In addition, CBCT has significantly lower dose in comparison to maxillofacial MDCT [5].

## 2. Case Presentation

**Figure 1:**
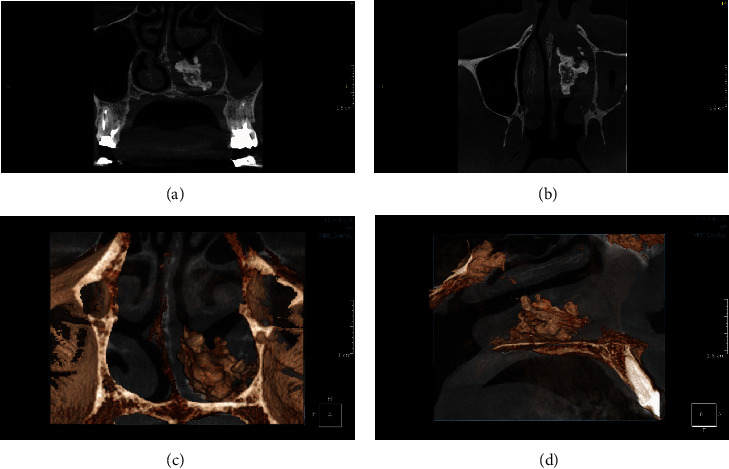
CBCT images of nasal fossa. Coronal (a), axial (b), and multiplanar reformat images show an irregularly large heterogenous calcified mass in the inferior meatus of the left nasal cavity. Note the adjacent mucosal thickening. Coronal (c) and sagittal (d) 3D reformation images better show the space occupying rhinolith and slight deviation of nasal septum to the right side.

A 40-year-old female was referred to a private oral and maxillofacial radiology clinic with recent two months history of facial and periorbital pain, headache, upper jaw pain, nasal discharge, epistaxis, and fetid odor sensation. The patient had no history of trauma or previous interventions. A medium FOV CBCT scan was acquired from maxilla and paranasal sinuses. The left inferior meatus was completely filled with a well-defined, irregular calcified mass with peripheral mucosal thickening (Figure 1). There was also mucosal thickening in the left maxillary sinus. The initial diagnosis was rhinolith. The patient was further referred to department of the ear, nose, and throat surgery, and the calcified mass was excised endoscopically under general anesthesia. The histopathologic examination confirmed a granulation tissue with nasal lithiasis pattern tissue. The left maxillary sinus also showed inflammatory mucosal thickening compatible with chronic sinusitis. The patient experienced immediate improvement, and postoperative follow-up of patient was uneventful. The area had healed with no complication.

## 3. Discussion

A rhinolith is a calcification around exogenous or endogenous nidus in the nasal cavities. Approximately, 15 years are needed for rhinoliths to form; therefore, they are initially asymptomatic [6]. Further calcification and expansion may impinge on the mucosa leading to pain, congestion, and ulceration. The common complications associated with this entity are nasal obstruction, septal erosion, blood stained rhinorrhea and epistaxis, sinusitis, headache, purulent discharge, and fetid odor sensation [2, 4, 7, 8]. However, the patient may not face any pain or symptoms at all [9]. The patient of this case complained from fetid odor sensation, dental and periorbital pain, and unilateral purulent discharge. Girgis et al. suggested assessment of nasal cavity for rhinoliths in the cases of the anterior maxillary teeth pain in the absence of odontogenic causes [2]. In this case, the ipsilateral maxillary sinus had some mucosal thickening which may be due to partial obstruction of the adjacent nasal cavity. If the rhinolith is large enough, it may cause complete obstruction of maxillary sinus ostium and further results in sinusitis [10].

Previous studies show that rhinolith is usually found in female in their third decade of life. However, it is also detected in younger ages [11, 12]. This case was also found in a 40-year-old female patient, which is consistent with previous studies. Mohiyuddin et al. reported a case of giant rhinolith in a 15-year-old girl [12]. The patient did not suffer from any facial pain or headaches, and the only symptom was mucopurulent nasal discharge.

In this case, the calcified entity was detected in the inferior meatus, where the passage is the narrowest in the nasal cavity. Rare locations for rhinolith have also been detected. Ersozlu et al. reported calcification and presence of rhinolith in a concha bullosa [4]. In this study, the patient suffered from nasal obstruction, snoring, and head ache, but did not have symptoms such as epistaxis. In the study of Kharoubi et al., unusual case of bilateral rhinolithiasis and destruction of the posterior nasal septum was reported [6].

Clinical examination of the patient may include nasal endoscopy, anterior rhinoscopy, and probing of the mass [12]. The clinical differential diagnosis may include chronic rhinosinusitis, especially fungal sinusitis due to the presence of calcification. if the rhinolith is highly calcified, it can be detected by plain radiographs; however, low-density rhinolith requires advanced imaging to be detected [4]. Anthroliths may also be confused with rhinolith in plain radiographic projections such as occlusal or lateral cephalometric view. In the study of Estrela et al., large reactional osteogenesis in the maxillary sinus associated with secondary root canal infection was detected using cone-beam computed tomographic (CBCT) imaging [13]. This reactional osteogenesis presents a radiopaque entity in the maxillary sinus and may also be included in the differential diagnosis. Lesions such as calcifying fibroma, chondroma, and chonrosarcoma and calcified polyps should be included in the possible differential diagnoses [8, 14]. In addition, radiopaque odontogenic lesions such as odontoma, osteoma, and ossifying fibroma should be ruled out. In these cases, three dimensional imaging such as cone-beam computed tomography can easily indicate the location, extension, and probable origin of the lesion.

## 4. Conclusion

In the absence of obvious clinical dental pathology, sinonasal cavity should be completely assessed for pathology, including the presence of rhinolith [15].

## Data Availability

The data used to support the findings of this study are available from the corresponding author upon request.
